# IL-8 as mediator in the microenvironment-leukaemia network in acute myeloid leukaemia

**DOI:** 10.1038/srep18411

**Published:** 2015-12-17

**Authors:** Alexander Kuett, Christina Rieger, Deborah Perathoner, Tobias Herold, Michaela Wagner, Silvia Sironi, Karl Sotlar, Hans-Peter Horny, Christian Deniffel, Heidrun Drolle, Michael Fiegl

**Affiliations:** 1 Department of Internal Medicine III, Klinikum der Universität München, Munich, Germany; 2 Institute of Pathology, Klinikum der Universität München, Munich, Germany

## Abstract

The bone marrow microenvironment is physiologically hypoxic with areas being as low as 1% O_2_, e.g. the stem cell niche. Acute myeloid leukaemia (AML) blasts misuse these bone marrow niches for protection by the local microenvironment, but also might create their own microenvironment. Here we identify IL-8 as a hypoxia-regulated cytokine in both AML cell lines and primary AML samples that is induced within 48 hours of severe hypoxia (1% O_2_). IL-8 lacked effects on AML cells but induced migration in mesenchymal stromal cells (MSC), an integral part of the bone marrow. Accordingly, MSC were significantly increased in AML bone marrow as compared to healthy bone marrow. Interestingly, mononuclear cells obtained from healthy bone marrow displayed both significantly lower endogenous and hypoxia-induced production of IL-8. IL-8 mRNA expression in AML blasts from 533 patients differed between genetic subgroups with significantly lower expression of IL-8 in acute promyelocytic leukaemia (APL), while in non APL-AML patients with FLT ITD had the highest IL-8 expression. In this subgroup, high IL-8 expression was also prognostically unfavourable. In conclusion, hypoxia as encountered in the bone marrow specifically increases IL-8 expression of AML, which in turn impacts niche formation. High IL-8 expression might be correlated with poor prognosis in certain AML subsets.

Recent evidence suggests that in acute myeloid leukaemia (AML) the microenvironment is an important part of disease progression and pathophysiology^1^. Exact mechanisms remain elusive, if nothing else due to the fact that the bone marrow is a complex tissue and this microenvironment comprises a multitude of different components existing in an intricate network (and as the name says: it is rather small). These components can be either cellular or non-cellular.

Cellular components are haematological (monocytes or blasts to name a few) or non-haematological cells (e.g. mesenchymal stromal cells [MSC]) residing in the bone marrow. MSC are a part of the stem cell niche, which is commonly referred to as *the* microenvironment in the context of AML. They are rather well understood, and they influence AML blasts in a variety of ways, e.g. by induction of metabolic changes^2^,^3^ or regulation of anti-apoptotic proteins^4^. Non-cellular components are even more diverse, and comprise of biological (e.g. cytokines) and physico-chemical features (e.g. O_2_). Due to the low solubility of O_2_ in tissue the bone marrow is hypoxic (i.e. ≤6% O_2_, depending on the region), which has been shown beyond doubt^5^,^6^,^7^. The functional impact of O_2_ however is less clear, but hypoxia has been shown to impact differentiation of normal haematological progenitors^8^ and expression/ phosphorylation of proteins in AML^5^,^6^,^9^.

While the effects of the microenvironment and parts thereof on AML have been extensively investigated, little attention has been paid to the effects AML exerts on the microenvironment. There are several studies of cytokine production of AML^10^,^11^, which can been considered a part of the microenvironment, but the biological consequences are unclear and are mostly considered as a mechanism for autocrine stimulation. Additionally, each part of the microenvironment has been looked at separately, which rather reflects a methodological problem than the underlying biology.

Hence, this project aimed to elucidate a factor that interlinks the cellular with the non-cellular microenvironment. This factor would most likely be a cytokine produced by AML, and to effectively combine the different components of the microenvironment, it would need to fulfil 2 requirements: (I) it has to be regulated by hypoxia and (II) in turn it should influence niche formation (i.e. MSC). Preliminary data suggested IL-8 (CXCL8) to be upregulated in AML by hypoxia, fulfilling the first prerequisite, while published data pointed on its influence on MSC (i.e. migration^12^), fulfilling the second requirement. Thus, it is an ideal candidate for further research.

## Results

### Presence of mesenchymal stromal cells is increased in AML bone marrow

**Figure 1:**
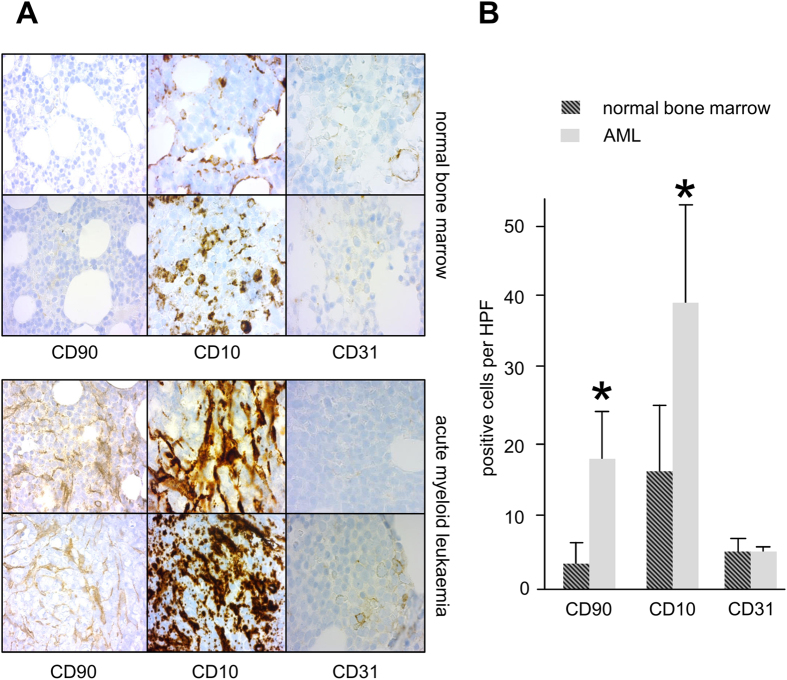
AML bone marrow has increased numbers of mesenchymal stromal cells as compared to normal bone marrow. (**A**) 2 representative samples each for normal bone marrow and AML (CD90, CD10 and CD31 staining, 630fold magnification). (**B)** Absolute numbers of CD90+ and CD10+ cells (MSC) were significantly increased in HPF (1000fold magnification) in AML bone marrow, while there was no difference for CD31+ cells (endothelial cells).

Mesenchymal stromal cells (MSC) are an integral part of the microenvironment in the bone marrow. Hence, in a first step we examined whether AML bone marrow differs in MSC presence from normal bone marrow. We investigated 8 random normal bone marrows and compared these to 8 random AML bone marrows for the staining of CD90+, CD10+ cells with a distinct morphology. As shown in Fig. 1, CD90+ and CD10+ cell density was visually markedly higher in AML bone marrow as compared to normal bone marrow (Fig. 1A: 2 representative samples each). CD90+ and CD10+ cells per high power field (HPF, 1000fold magnification, 2 replicates each) were also significantly higher in AML samples (Fig. 1B: CD90+: 19.0 vs. 3.5, p < 0.01, CD10+: 39.0 vs. 18.6, p < 0.05).There was no significant difference in CD31+ cells (4.2 vs. 4.2, p = 0.19, Fig. 1A and B), a marker for endothelial cells, which are also positive for CD90. Hence, the CD90+ cells increased in AML samples are not endothelial cells. As samples were fully anonymized, no clinical data was available for these patients.

### Regulation of cytokines by hypoxia in AML

**Figure 2:**
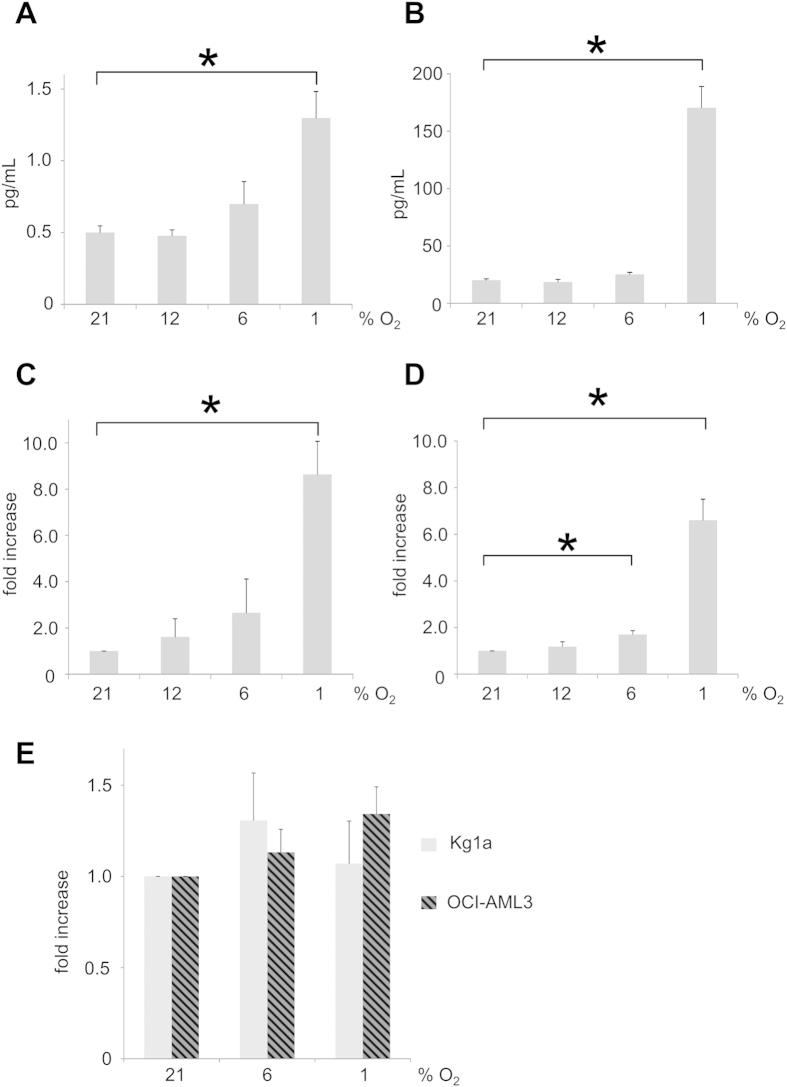
Hypoxia mediated cytokine secretion in AML cell lines. (**A**) In Kg1a, only profound hypoxia of 1% O_2_ significantly increases IL-8 secretion after 48 hours as compared to 21% O_2_. (**B**) Similar results were obtained in OCI-AML3 cell line. (**C**) Kg1a cells adapted to hypoxia for 8 days maintain increased secretion of IL-8. (**D**) Il-8 secretion of hypoxia adapted OCI-AML3 cells. (**E**) No significant increase of HGF production in both AML cell lines.

As AML blasts and MSC might interact via cytokines, we performed a screening assay for 80 cytokines in the supernatant of AML cell lines. In this screening, only IL-8 and HGF showed a significant (semi quantitative) increase at hypoxic conditions and were therefore further investigated (data not shown). As shown in Fig. 2, IL-8 and HGF secretion was measured during different time points (2 and 10 days) and at different oxygen levels (21%, 12%, 6% and 1%) in AML cell lines Kg1a and OCI-AML3. Here we found that, despite significant differences in IL-8 production between the cell lines (Kg1a 0.5 pg/mL versus OCI-AML3 20.1 pg/mL [p < 0.01, mean standardized to 10^5^ viable cells/mL]) after 48 hours IL-8 secretion was significantly increased in both cell lines at 1% O_2_ only, while the effects of both 12% and 6% O_2_ did not differ from IL-8 secretion at standard laboratory conditions of 21% O_2_ (increase as compared to 21% O_2_: Kg1a: 12% 0.96fold [n.s.], 6% 1.48fold [n.s.], 1% 2.61fold [p < 0.05]; OCI-AML3 12% 0.92fold [n.s.], 6% 1.27fold [n.s.], 1% 8.62fold [p < 0.01]). Similarly, both cell lines maintained this increased production of IL-8 even when adapted to the respective level of hypoxia for up to 10 days. We also observed that at 6% O_2_ IL-8 production was already slightly increased in OCI-AML3 (pg/mL per 10^5^ cells; Kg1a 21% 0.76, 12% 1.14 [n.s.], 6% 1.86 [n.s.], 1% 6.57 [p < 0.05]; OCI-AML3 21% 15.11 12% 17.83 [n.s.], 6% 27.70 [p < 0.05], 1% 99.37 [p < 0.01]). However, we were unable to detect any differences for HGF and hence abandoned further investigation of this cytokine, focusing on IL-8 instead for further experiments.

### Hypoxia induced expression of IL-8 in AML

**Figure 3:**
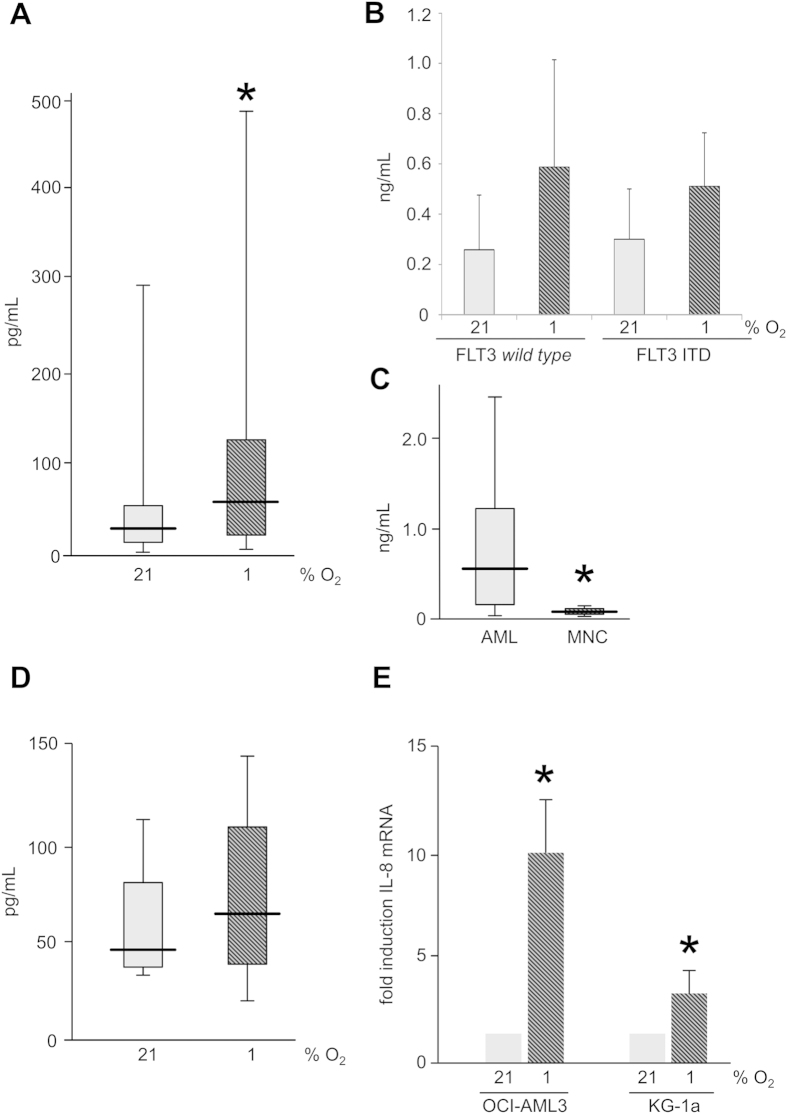
Hypoxia induced expression of IL-8 in AML. (**A**) In primary samples of patients with AML (n = 22), hypoxia increases secretion of IL-8 by 2fold. (**B**) FLT3 mutational status did not influence IL-8 secretion. (**C**) Normal hematopoietic progenitors (MNC mononuclear cells) produce significantly less IL-8 at hypoxic conditions than AML. (**D**) Healthy hematopoietic progenitors do not induce IL-8 at hypoxia. (**E**) Hypoxia increases IL-8 mRNA in AML cell lines OCI-AML3 and KG-1a.

Next, we confirmed the results of AML cell lines in primary AML samples. For these experiments, a time point of 48 hours and 1% O_2_ were chosen based on the previous results. Patient samples from 22 patients with AML were available (median age 69 [22–82] years, male 45%). IL-8 secretion was profoundly higher in primary AML samples as compared to AML cell lines (after 48 hours: 7.8 ± 9.1 [for Kg1a and OCI-AML3] versus 459.1 ± 644.4 pg/ml, p < 0.01), and induction of IL-8 expression at hypoxia of 1% O_2_ was also confirmed in the primary samples (2fold, p < 0.01: Fig. 3A).

Next, we performed subgroup analyses concerning differential expression and hypoxia induced induction of IL-8 in genetically defined AML patients. FLT3 wild type and FLT3-ITD patients did not differ significantly at their baseline secretion of IL-8 at 21% O_2_ (258.5 pg/ml per 10^5^ FLT3 wild type cells versus 300.1 pg/ml per 10^5^ FLT3 ITD cells) nor did they differ in hypoxia mediated induction of IL-8 (2.3fold versus 1.7fold, Fig. 3B). Similar results were obtained for NPM1 (250.2 pg/ml and 2.9fold induction by hypoxia, not significant). Interestingly, secretion of IL-8 of FICOLL density centrifugation enriched mononuclear cells from the bone marrow of healthy individuals (n = 4) was significantly lower than that of AML blasts after 48 hours at 1% O_2_ *in vitro* (548.0 pg/ml versus 62.8 pg/ml, p < 0.01 [Mann-Whitney-test], Fig. 3C). In addition, these healthy hematopoietic progenitors showed no significant increase of IL-8 under hypoxia (p = 0.17, Fig. 3D). Hypoxia induced increase of IL-8 secretion was due to increased translational processing, as IL-8 PCR revealed significantly increased levels of mRNA for IL-8 after 48 hours of 1% O_2_ as compared to 21% O_2_ in AML cell lines OCI-AML3 and KG-1a (ΔΔCT 3.50 for OCI-AML3 and 1.95 for KG-1a, p < 0.01; Fig. 3E).

### Effects of IL-8 on AML and the microenvironment

**Figure 4:**
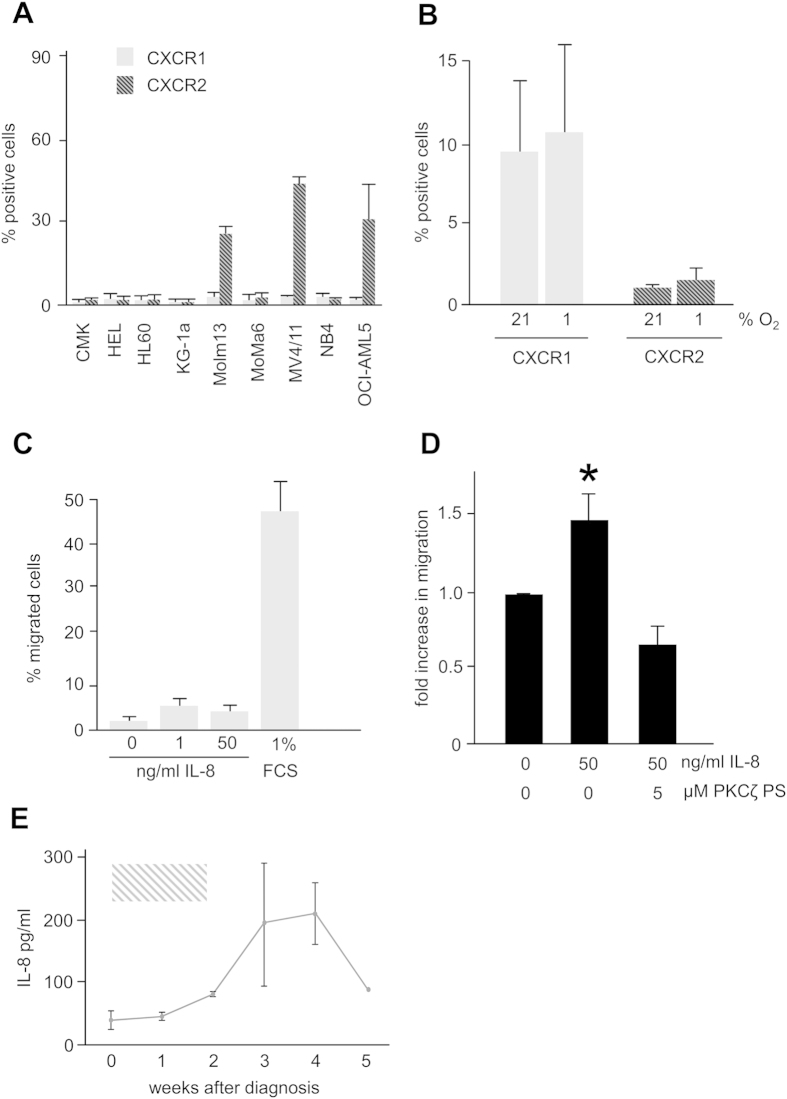
Effects of IL-8 in AML and on the microenvironment. (**A**) While IL-8 receptor CXCR1 is not expressed on 9/9 AML cell lines, in 3/9 cell lines IL-8 receptor CXCR2 is expressed on 26.5–49.6% of cells. (**B**) App. 10% of primary MSC express IL-8 receptor CXCR1 on their surface but no IL-8 receptor CXCR2. Expression is independent from pO_2_. (**C**) MV4/11 cells do not migrate against IL-8 in transwell experiments (1% FCS: positive control). (**D**) Primary bone marrow derived MSC (n = 3) however do significantly migrate towards an IL-8 gradient. This migration can be abrogated by inhibition of PKCζ. (**E**) IL-8 serum levels in patients with AML undergoing intensive chemotherapy (grey bar: period of induction chemotherapy with sequential high dose cytarabine and mitoxantrone).

We next examined the effects of IL-8 on AML blasts for possible autocrine mechanisms. First, expression of IL-8 receptor alpha (CXCR1) and beta (CXCR2) on AML cell lines was investigated. CXCR1, considered more specific for IL-8 was absent on all 9 cell lines (CMK, HEL, HL60, Kg-1a, Molm13, MonoMac6, MV4/11, NB4, OCI-AML5) investigated, and “promiscuous” CXCR2 (also having affinity for CXCL1, 2, 3, 5, 6, 7 and 8) was also absent on the majority (6 out of 9) of these cell lines (Fig. 4A) and hypoxia did not affect the expression of both IL-8 receptors (data not shown). We then investigated whether IL-8 could have an impact on the cellular microenvironment instead of auto- or paracrine stimulation and found that out of all primary MSC investigated (n = 4, 2 from healthy volunteers and 2 from AML patients) a median of 9.7% of cells expressed IL-8 receptor CXCR1 on their surface, independently of the oxygen state they were in (increase 1.15fold in % positive cells, Fig. 4B). In addition, CXCR1 and CXCR2 mRNA expression (median 9.17 ± 0.38 versus 6.15 ± 0.85, p < 0.001) in 533 AML patients (for details, see section 3.5), while highly correlated with each other (r = 0.43, p < 0.001 [Pearson]) had no impact on overall (Hazard ratios [HR]: CXCR1 0.91 [95 CI 0.68–1.21], CXCR2 HR 0.96 [0.84–1.09]) and relapse free survival (HR: CXCR1 0.96 [0.65–1.4], CXCR2 1.02 [0.87–1.21]). Also, median expression did not separate prognostic groups as described previously (median OS for CXCR1 316 days (≤median) versus 374 days (>median) [n.s.], for CXCR2 342 days (≤median) versus 344 days (>median) [n.s.]).

According to this low to absent expression of IL-8 receptors in AML, cell line MV4/11 (displaying the highest CXCR2 expression) did not migrate towards increasing doses of chemokine IL-8 in transwell experiments (Fig. 4C, similar results for Molm13, Kg1a and OCI-AML3 are not shown). Instead, primary MSC from the bone marrow migrated towards IL-8 (1.45 fold, p < 0.01, Fig. 4D). These data implicate an effect of IL-8 on MSC rather than the myelopoiesis. Interestingly, migration of MSC could be abrogated by inhibition of protein kinase C zeta (PKCζ) by 5 μM of PKCζ pseudosubstrate.

However, when serum levels of IL-8 in patients with AML (n = 4 with at least 3 serum specimen at 1 time point available) that were undergoing intensive chemotherapy (with sequential high-dose AraC and mitoxantrone as induction chemotherapy^13^ were examined, it was interesting to observe that at initial diagnosis with high leukemic burden and granulocytopenia, serum levels of IL-8 were lowest (40 pg/mL), increasing during aplasia (maximum of 210 pg/mL after 4 weeks) and then decreasing to lower levels after achievement of CR and recovery of peripheral blood count (88 pg/mL, Fig. 4E).

### Clinical impact of IL-8 RNA expression

In addition, we analysed the expression of IL-8 in leukemic blasts from 533 AML patients that were treated within the AMLCG1999 trial^14^. For details of trial treatment, please refer to the publication. Patients of the cohort investigated had an average age of 55.2 years (18–85 years), 50.3% of them were male. Of these patients, 26.5% were grouped into the favourable subgroup according to European Leukemia Net^15^, 20.8% into the intermediate I and 20.1 into the intermediate II subgroup. 25.1% belonged to the unfavourable subgroup. In 7.5% classification according to ELN was not possible. In 126 patients (23.6%) a FLT-ITD mutation was found. 289 patients (54.0%) achieved a complete remission. In addition, we had RNA expression and clinical data from 29 patients with acute promyelocytic leukaemia (APL). The median age of this cohort was 51 years (21 – 83 years), 12 patients were male and CR was achieved in 81%.

**Figure 5:**
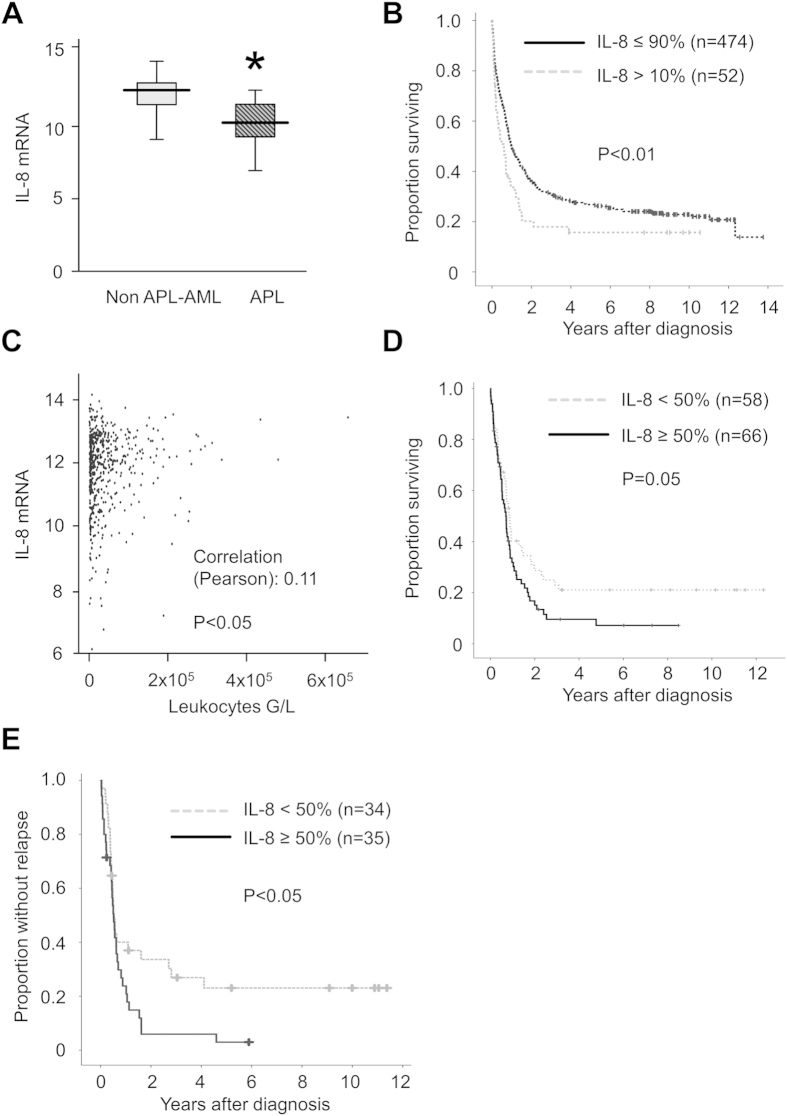
Clinical impact of IL-8 mRNA expression in AML. (**A**) Significant lower (semi-quantitative) levels of IL-8 mRNA in APL cells as compared to non APL-AML cells (p < 0.01). (**B**) IL-8 mRNA expression in AML blasts above the 10^th^ percentile confers a significant worse OS in AML patients. (**C**) IL-8 mRNA levels were significantly correlated with peripheral leukocyte count at initial diagnosis. (**D**) In FLT3-ITD patients (n = 124), IL-8 mRNA levels above the median tend to confer a decreased survival (borderline significance). (**E**) For RFS this cut-off value significantly divided the FLT3-ITD population.

In the full cohort of patients (AML and APL patients combined), higher levels of IL-8 were found to profoundly impact overall survival (OS) and relapse free survival (RFS). Hazard ratio (HR) was 1.11 (1.03–1.20, p < 0.01) for OS and 1.13 (1.02–1.26, p < 0.05) for RFS. However, this effect was lost when only non APL-AMLs were analysed (HR: OS: 1.04 [0.96–1.14], n.s., RFS 1.04 [0.93–1.17], n.s.), the reason being a significant lower expression of IL-8 in APL than non APL-AML (Fig. 5A). However, the 10% patients with non APL-AML that displayed the highest expression (cut-off 13.08) did still have a shorter OS (median 5.7 months vs. 12.0 months, p < 0.01 [log-rank], Fig. 5B). This effect might be attributable to the significant association of IL-8 mRNA with peripheral leukocyte count (a well-established prognostic factor) at initial diagnosis (Fig. 5C) or differential expression of IL-8 in prognostic subgroups. We therefore analysed the expression of mRNA IL-8 expression of leukemic blasts in different genetically defined subgroups of non APL-AML patients. Interestingly, while there was no difference in IL-8 mRNA between NPM1 wild type (n = 178; 11.84 ± 1.2,) and NPM1 mutated samples (n = 116, 12.1 ± 1.0, not significant), the presence of a FLT3-ITD (n = 126) was associated with increased mRNA expression in the AML blasts (12.1 ± 1.0 versus 11.8 ± 1.3 for FLT wild type [n = 396], p < 0.05). Next, we analysed whether IL-8 expression had any particular effect according to FLT3 mutational status. While there was no effect on survival parameters in the FLT3 wild type cohort (n = 396), using Cox regression a significant impact of IL-8 expression was noted within the FLT3-ITD group (n = 124) on OS (HR 1.34 [1.01–1.78], p < 0.05) and RFS (HR 1.49 [1.02–2.19], p < 0.05). As a cut-off we chose the median of IL-8 mRNA in this group, and found that this threshold was predictive for OS (Fig. 5D, borderline significance) and RFS (Fig. 5E).

## Discussion

The microenvironment of the bone marrow emerges as a critical player in cancer biology. However, mostly isolated parts of the microenvironment have been investigated, in the case of AML e.g. either MSC^4^,^16^ or bone marrow hypoxia^5^,^6^ and their effects on AML blasts. In this study, we tried to identify components that link different parts of the microenvironment and also AML to each other and found IL-8 (CXCL8) to be such a possible missing link.

CXCL8 has long been identified as a pro-inflammatory cytokine and a strong chemoattractant for neutrophils^17^. Besides this physiological function, environmental stresses, such as hypoxia, acidosis, chemotherapy, etc. have been attributed to induction of IL-8 in tumour tissue^18^. In addition, constitutional elevated levels of CXCL8 have been observed various cancers, e.g. prostate^19^, colorectal^20^ and non-small cell lung cancer^21^. Two receptors for CXCL8 have been identified, the more specific CXCR1 and the more “promiscuous” CXCR2 that also binds other CXC ligands (1, 2, 3, 5, etc.)^18^. After ligand binding of these G-protein-coupled receptors, a range of intracellular signalling pathways are activated, including MAPK, PI3K, PKC, FAK and Src^18^, which confer at least in part to diminished apoptosis and increased proliferation. Indeed, CXCL8 was shown to induce chemo resistance against oxaliplatin and also etoposide in solid tumours^18^.

However, little is known about the role of IL-8 in AML. The overexpression of the chemokine in AML has been known for over 20 years^22^ and has recently been confirmed^23^. We also found IL-8 to be constantly expressed in AML, albeit with differences in genetic defined subtypes: we found the lowest expression of mRNA in APL and the highest expression in AML with FLT-ITD – which might also explain prognostic differences associated with IL-8 expression. However, we were unable to observe differences in IL-8 protein secretion *in vitro* by different AML subgroups, but this is most likely owed to the fact that the number of patient samples was too small and hence underpowered for the detection of this difference. In addition, we found that IL-8 mRNA in AML blasts correlated to initial blast count, an old but well established risk factor^24^,^25^ and IL-8 was of prognostic significance in the cohort of patients harbouring a FLT-ITD mutation. But if we assume a general biological role for IL-8 in AML, maybe even a *conditio sine qua non*, it is not quite so surprising that no strong impact on prognosis is observed as potentially all AML are dependent on it. The course of serum levels of IL-8 in AML patients - correlating with leukocyte burden (but not with AML blasts) - suggests however that IL-8 is not solely produced by AML but rather related to healthy myelopoiesis. However, questions remain concerning which cells actually produce this serum IL-8 *in vivo* and whether there are differences in IL-8 concentrations between the bone marrow and the peripheral blood. It should of course not be forgotten that IL-8 is an inflammatory cytokine in the first place, and hence this increase might simply reflect the fact that patients in aplasia after chemotherapy suffer from infections due to neutropenia, which are known to increase IL-8 levels^26^,^27^.

The induction of IL-8 by hypoxia has been described in solid tumours^28^, and IL-8 is indeed considered a downstream target of HiF1α^29^. This increase by hypoxia has always been attributed to environmental stress, but our data do not support this notion in the case of AML. Hypoxia of 1% O_2_ is considered physiological in the stem cell niche^8^, and the whole bone marrow is considered to be hypoxic^5^. The investigated cells were not stressed functionally by this low oxygen level (no impaired viability) and IL-8 was the only cytokine upregulated by hypoxia and the upregulation was quite specifically time and dose dependent. As expected, IL-8 expression was transcriptionally regulated by hypoxia. Interestingly, we observed a significant difference between normal mononuclear bone marrow cells and AML blasts: normal cells expressed significantly lower IL-8 protein and did not show an upregulation by hypoxia.

The role of IL-8 receptors remain elusive: one recent publication found CXCR2 to be overexpressed in AML on a mRNA level, but did not show direct expression by e.g. flow cytometry or microscopy. They additionally found a significantly higher expression of CXCR2 mRNA than CXCR1 mRNA and also a prognostic impact in 200 AML samples^23^. We however were unable to confirm these findings on our much larger cohort of 533 patients. Median CXCR1 mRNA was significantly higher in our cohort than CXCR2 and neither had any prognostic impact. We also found actual CXCR2 expression measured by FACS in only 30% of AML cell lines. While we can only explain these differences by different methods (mRNA versus flow cytometry) and different AML patient collectives, based on our data we would like to propose a different mechanism for IL-8 in the context of AML: no auto- or parakrine stimulation of AML but instead, modulation of the microenvironment by AML blasts; and while a proangiogenic effect of IL-8 – angiogenesis being microenvironment as well–has been well established^30^, in our hypothesis, the affected microenvironment would be mesenchymal stromal cells.

Indeed, we found that bone marrow derived MSC (I) expressed CXCL1 but not CXCL2 and that (II) primary MSC migrated towards an IL-8 gradient *in vitro*, confirming previous reports^12^. Now, given that the production of IL-8 by AML blasts (but not normal mononuclear cells from the bone marrow) is stimulated by the pO_2_ present in the stem cell niche one would assume that MSC density in AML bone marrow is increased when compared to normal bone marrow and this is exactly what we observed. The effects of MSC on AML have been well established meanwhile and need not be reviewed here again in detail. They are mostly chemo-protective, e.g. by upregulation of anti-apoptotic proteins^4^ or metabolic changes^2^,^3^.

As the CXCL8-IL8 receptor axis has been identified as an drugable target in e.g. diseases with chronic inflammation, several mechanisms have been developed to inhibit it. One way is direct inhibition via neutralizing antibodies for CXCL8, which were mainly developed for the treatment of inflammatory diseases like chronic obstructive pulmonic disease, but which also did show promising results in pre-clinical melanoma models^31^. In addition, small molecules have been developed that antagonize CXCR1/2 signalling. Here, CXCR1 blockade by reparixin has shown promising activity against breast cancer in mouse xenografts models^32^ and CXCR1/2 inhibition by SCH-527123 did reduce tumour growth and microvessel density in a xenograft model of colorectal cancer^33^. While clinical data about the anti-cancer efficacy of these drugs are missing, the data available from clinical trials have proven the safety and feasibility of these drugs^18^.

In conclusion, this data supports the importance of IL8 in AML but provides an additional novel mechanism: IL-8 could be the missing link that ties MSC and hypoxia in AML together. As the means of inhibition of the CXCL8-IL-8 receptor axis are at hand, further studies on the therapeutic intervention seem warranted and may utilize a novel concept in AML therapy: to aim at the crucial AML microenvironment interaction rather than the leukemic cell itself.

## Methods

### Immunohistochemistry of bone marrow biopsies

Immunohistochemical analyses were performed on archival bone marrow trephine biopsies taken from the iliac crest after informed consent had been obtained from the patients. Diagnosis of AML was confirmed according to WHO criteria (≥20% myeloid blasts with >3% positivity for peroxidase). Eight samples of patients with AML were used; these samples were completely anonymized for analysis, hence no further information on these patients is available. Eight normal controls consisted of patients without bone marrow involvement; again, these patients were anonymized and no further information was made available. After formalin-fixation, these specimens had been mildly decalcified overnight in acetic acid, and embedded in paraffin wax. Sections were cut at 3 *μ*m and stained for CD90 (Acris, San Diego, USA), CD10 (Ventana Medical Systems, Tucson, AZ, USA) and CD31 (Dako, Hamburg, Germany) by the indirect immunoperoxidase-staining technique as described elsewhere^34^.

### Cell culture

Standard laboratory (normoxic) conditions comprised 21% O_2_, 5% CO_2_, and 37 °C. For experiments in a reduced oxygen environment, the hypoxic Workstation INVIVO_2_ 400 from Ruskinn Technology (Bridgend, United Kingdom) was used. Cells were incubated at 12%, 6% or 1% O_2_, 5% CO_2_, and 37 °C. Live cell numbers were evaluated by trypane blue exclusion (Life Technologies, Darmstadt, Germany).

AML cell lines were all obtained from the DSMZ (German collection of Microorganisms and Cell Cultures, Braunschweig, Germany). Cell lines were maintained at a density of 2.0 × 10^5^/ml in RPMI 1640 containing 10% FCS (Biochrom, Berlin, Germany), 2 mM glutamine and 1% penicillin-streptomycin (all from Life Technologies, Darmstadt, Germany). Primary AML samples were collected from bone marrow aspirates or from peripheral blood from AML patients who underwent routine diagnostics. Mononuclear cells were separated by Ficoll-Hypaque (Sigma-Aldrich, St Louis, MO) density-gradient centrifugation and cultured at a density of 10^6^/ml in RPMI 1640 containing 10% FCS, 2 mM glutamine and 1% penicillin-streptomycin. Experiments were performed on fresh and frozen cells. All experiments were carried out in accordance with approved guidelines and regulations. All experimental procedures were approved by the local ethics committee (Medical Faculty of the Ludwig-Maximilians-Universität, Munich, Germany).

### Enzyme-linked immunosorbent assay (ELISA) and cytokine array

Supernatant of primary AML cells (10^6^/ml) or AML cell lines KG-1a and OCI-AML-3 (2.0 × 10^5^/ml) were collected after 48 h of *in vitro* cultivation at the indicated oxygen concentration by centrifugation (10 min, 2000 rpm). For long term cultures cell lines were pre-cultured under hypoxic conditions for 8 days prior to this treatment. IL-8 and HGF protein in the supernatant was quantified with the human IL-8 ELISA Kit II (BD Biosciences, San Jose, USA) and human HGF ELISA (R&D Systems, Minneapolis, USA) according to the manufacturer’s instructions. For quantification of IL-8 protein in patients with AML, serum samples were collected during routine surveillance at the indicated time points and spun down at 2000 rpm for 10 minutes. Serum was stored at −80 °C and IL-8 quantification was performed by ELISA as described.

### Flow cytometry

Primary AML cells or AML cell lines were cultured at the respective conditions, collected by centrifugation after the indicated time points and stained with anti-human fluorescence labelled antibodies CXCR1 APC and CXCR2 FITC (all from BD Biosciences, San Jose, USA) according to the manufacturer’s instructions with appropriate isotype controls. Flow cytometry was performed with a FACSCalibur cytometer (BD Biosciences, San Jose, USA) and data was analysed with BD CellQuest software.

### IL-8 PCR

AML cell lines KG-1a and OCI-AML-3 were collected after 5 days of culture at the appropriate oxygen conditions and total RNA was extracted with the Absolutely RNA Mini Prep Kit (Agilent Technologies, Santa Clara, USA) following the manufacturer’s instructions. For RT-PCR the First-Strand cDNA Synthesis System (Life Technologies, Carlsbad, USA) with the supplied oligo-(dT)_20_ primer was applied. Quantitative PCR amplifications were performed using Fast SYBR Green Master Mix (Applied Biosystems, Waltham, USA) on the 7900HT Fast Real Time PCR system from the same company. For IL-8 cDNA amplification the following primers were used: human IL-8 forward 5′-TAGCAAAATTGAGGCCAAGG-3′ and reverse 5′-AGCAGACTAGGGTTGCCAGA-3′ (Biomol GmbH, Hamburg Germany). ß-actin was used as housekeeping gene: human ß-actin forward 5′-CCGAGGACTTTGATTGCACA-3′ and reverse 5′-AGTGGGGTGGCTTTTAGGAT-3′. (MWG Biotech, Ebersberg Germany). PCR conditions were: 95 °C for 15 min followed by 40 cycles of 95 °C for 15 sec, 60 °C for 30 sec, and 72 °C for 30 sec. Fold of RNA induction relative to normoxic conditions was calculated as the mean 2e^ΔΔCt^ of n = 4 independent experiments.

### Migration assay

Migration of cell lines was assessed in Transwell permeable 24 well plates with 5.0 μM pores (Costar, Washington USA). The lower compartment contained 500 μL of serum free media supplemented with indicated concentrations of IL-8 (R&D Systems, MN, USA), while 10^5^ cells were placed in the upper compartment. After 24 hrs the amount of viable cells migrated to the lower compartment was determined by trypan blue exclusion cell counting in at least three independent experiments. Migration of MSCs was assessed in Transwell permeable 96 well plates with 8.0 μM pores using the fluorimetric QCM Cell Migration Assay Kit (Millipore, Billerica MA, US). The lower compartment contained 150 μL of serum free media supplemented with IL-8. 5 × 10^4^ primary MSC were applied to the upper compartment and allowed to migrate for 16 hrs. The amount of migrated cells was determined according to the manufactures instructions at 480/520 nm using Cytofluor 4000 Fluorescence/Bioluminescence Plate Reader (Applied Biosystems, Waltham, USA). MYR PKCζ pseudosubstrate was from Life Technologies (Darmstadt, Germany).

### Microarray analyses

Pre-treatment bone marrow samples of 562 patients at time of diagnosis were analysed using Affymetrix U133A + B and Affymetrix U133 Plus2.0 microarrays (Affymetrix, Santa Clara, CA) as published previously^35^. The microarray data have been deposited in the Gene Expression Omnibus with the accession number GSE37642^36^. Expression data are presented as log2 throughout the study.

### Statistical analysis

Results are shown as the mean ± SEM or SD of at least 3 experiments each. Paired data were analysed using the paired Student *t* test. If other tests were used, they are specified at the according section. For survival analyses, Cox regression and the method of Kaplan-Meier using Log Rank test with SPSS Statistics® (Version 22, IBM) were used. Microsoft Excel was used for data acquisition and storage. A *P* value < 0.05 was considered statistically significant.

## Additional Information

**How to cite this article**: Kuett, A. *et al.* IL-8 as mediator in the microenvironment-leukaemia network in acute myeloid leukaemia. *Sci. Rep.* **5**, 18411; doi: 10.1038/srep18411 (2015).

## References

[b1] Liersch R., Muller-Tidow C., Berdel W. E. & Krug U. Prognostic factors for acute myeloid leukaemia in adults–biological significance and clinical use. Br J Haematol 165, 17–38 (2014). 24484469 10.1111/bjh.12750

[b2] Samudio I., Fiegl M. & Andreeff M. Mitochondrial uncoupling and the Warburg effect: molecular basis for the reprogramming of cancer cell metabolism. Cancer Res 69, 2163–2166 (2009). 19258498 10.1158/0008-5472.CAN-08-3722 PMC3822436

[b3] Samudio I. *et al.* Pharmacologic inhibition of fatty acid oxidation sensitizes human leukemia cells to apoptosis induction. J Clin Invest 120, 142–156 (2010). 20038799 10.1172/JCI38942 PMC2799198

[b4] Konopleva M. *et al.* Stromal cells prevent apoptosis of AML cells by up-regulation of anti-apoptotic proteins. Leukemia 16, 1713–1724 (2002). 12200686 10.1038/sj.leu.2402608

[b5] Fiegl M. *et al.* CXCR4 expression and biologic activity in acute myeloid leukemia are dependent on oxygen partial pressure. Blood 113, 1504–1512 (2009). 18957686 10.1182/blood-2008-06-161539 PMC2644078

[b6] Fiegl M. *et al.* Physiological hypoxia promotes lipid raft and PI3K-dependent activation of MAPK 42/44 in leukemia cells. Leukemia 24, 1364–1367 (2010). 20508615 10.1038/leu.2010.94 PMC4105972

[b7] Parmar K., Mauch P., Vergilio J. A., Sackstein R. & Down J. D. Distribution of hematopoietic stem cells in the bone marrow according to regional hypoxia. Proc Natl Acad Sci USA 104, 5431–5436 (2007). 17374716 10.1073/pnas.0701152104 PMC1838452

[b8] Abdel-Wahab O. & Levine R. L. Metabolism and the leukemic stem cell. J Exp Med 207, 677–680 (2010). 20368582 10.1084/jem.20100523 PMC2856035

[b9] Drolle H. *et al.* Hypoxia regulates proliferation of acute myeloid leukemia and sensitivity against chemotherapy. Leuk Res 39, 779–785 (2015). 25982178 10.1016/j.leukres.2015.04.019

[b10] Kornblau S. M. *et al.* Recurrent expression signatures of cytokines and chemokines are present and are independently prognostic in acute myelogenous leukemia and myelodysplasia. Blood 116, 4251–4261 (2010). 20679526 10.1182/blood-2010-01-262071 PMC4081283

[b11] Kittang A. O., Hatfield K., Sand K., Reikvam H. & Bruserud O. The chemokine network in acute myelogenous leukemia: molecular mechanisms involved in leukemogenesis and therapeutic implications. Curr Top Microbiol Immunol 341, 149–172 (2010). 20376612 10.1007/82_2010_25

[b12] Picinich S. C., Glod J. W. & Banerjee D. Protein kinase C zeta regulates interleukin-8-mediated stromal-derived factor-1 expression and migration of human mesenchymal stromal cells. Exp Cell Res 316, 593–602 (2010). 19944094 10.1016/j.yexcr.2009.11.011

[b13] Braess J. *et al.* Dose-dense induction with sequential high-dose cytarabine and mitoxantone (S-HAM) and pegfilgrastim results in a high efficacy and a short duration of critical neutropenia in *de novo* acute myeloid leukemia: a pilot study of the AMLCG. Blood 113, 3903–3910 (2009). 19131552 10.1182/blood-2008-07-162842

[b14] Buchner T. *et al.* Double induction containing either two courses or one course of high-dose cytarabine plus mitoxantrone and postremission therapy by either autologous stem-cell transplantation or by prolonged maintenance for acute myeloid leukemia. J Clin Oncol 24, 2480–2489 (2006). 16735702 10.1200/JCO.2005.04.5013

[b15] Dohner H. *et al.* Diagnosis and management of acute myeloid leukemia in adults: recommendations from an international expert panel, on behalf of the European LeukemiaNet. Blood 115, 453–474 (2010). 19880497 10.1182/blood-2009-07-235358

[b16] Samudio I., Fiegl M., McQueen T., Clise-Dwyer K. & Andreeff M. The warburg effect in leukemia-stroma cocultures is mediated by mitochondrial uncoupling associated with uncoupling protein 2 activation. Cancer Res 68, 5198–5205 (2008). 18593920 10.1158/0008-5472.CAN-08-0555 PMC2562568

[b17] Matsushima K. *et al.* Molecular cloning of a human monocyte-derived neutrophil chemotactic factor (MDNCF) and the induction of MDNCF mRNA by interleukin 1 and tumor necrosis factor. J Exp Med 167, 1883–1893 (1988). 3260265 10.1084/jem.167.6.1883 PMC2189694

[b18] Campbell L. M., Maxwell P. J. & Waugh D. J. Rationale and Means to Target Pro-Inflammatory Interleukin-8 (CXCL8) Signaling in Cancer. Pharmaceuticals 6, 929–959 (2013). 24276377 10.3390/ph6080929 PMC3817732

[b19] Murphy C. *et al.* Nonapical and cytoplasmic expression of interleukin-8, CXCR1, and CXCR2 correlates with cell proliferation and microvessel density in prostate cancer. Clin Cancer Res 11, 4117–4127 (2005). 15930347 10.1158/1078-0432.CCR-04-1518

[b20] Li A., Varney M. L. & Singh R. K. Expression of interleukin 8 and its receptors in human colon carcinoma cells with different metastatic potentials. Clin Cancer Res 7, 3298–3304 (2001). 11595728

[b21] Yuan A. *et al.* Interleukin-8 messenger ribonucleic acid expression correlates with tumor progression, tumor angiogenesis, patient survival, and timing of relapse in non-small-cell lung cancer. Am J Respir Crit Care Med 162, 1957–1963 (2000). 11069840 10.1164/ajrccm.162.5.2002108

[b22] Tobler A. *et al.* Constitutive expression of interleukin-8 and its receptor in human myeloid and lymphoid leukemia. Blood 82, 2517–2525 (1993). 8400299

[b23] Schinke C. *et al.* IL8-CXCR2 pathway inhibition as a therapeutic strategy against MDS and AML stem cells. Blood 125, 3144–3152 (2015). 25810490 10.1182/blood-2015-01-621631 PMC4432009

[b24] Pastore F. *et al.* Combined molecular and clinical prognostic index for relapse and survival in cytogenetically normal acute myeloid leukemia. J Clin Oncol 32, 1586–1594 (2014). 24711548 10.1200/JCO.2013.52.3480 PMC4876345

[b25] Schwartz R. S., Mackintosh F. R., Halpern J., Schrier S. L. & Greenberg P. L. Multivariate analysis of factors associated with outcome of treatment for adults with acute myelogenous leukemia. Cancer 54, 1672–1681 (1984). 6592033 10.1002/1097-0142(19841015)54:8<1672::aid-cncr2820540831>3.0.co;2-f

[b26] Schwaighofer H. *et al.* Serum levels of interleukin 6, interleukin 8, and C-reactive protein after human allogeneic bone marrow transplantation. Transplantation 58, 430–436 (1994). 8073511 10.1097/00007890-199408270-00007

[b27] Tilg H. *et al.* Interleukin-8 serum concentrations after liver transplantation. Transplantation 53, 800–803 (1992). 1314439 10.1097/00007890-199204000-00019

[b28] Shi Q., Xiong Q., Le X. & Xie K. Regulation of interleukin-8 expression by tumor-associated stress factors. J Interferon Cytokine Res 21, 553–566 (2001). 11559433 10.1089/10799900152547812

[b29] Kim K. S., Rajagopal V., Gonsalves C., Johnson C. & Kalra V. K. A novel role of hypoxia-inducible factor in cobalt chloride- and hypoxia-mediated expression of IL-8 chemokine in human endothelial cells. J Immunol 177, 7211–7224 (2006). 17082639 10.4049/jimmunol.177.10.7211

[b30] Strieter R. M., Belperio J. A., Phillips R. J. & Keane M. P. CXC chemokines in angiogenesis of cancer. Semin Cancer Biol 14, 195–200 (2004). 15246055 10.1016/j.semcancer.2003.10.006

[b31] Huang S. *et al.* Fully humanized neutralizing antibodies to interleukin-8 (ABX-IL8) inhibit angiogenesis, tumor growth, and metastasis of human melanoma. Am J Pathol 161, 125–134 (2002). 12107097 10.1016/S0002-9440(10)64164-8 PMC1850702

[b32] Ginestier C. *et al.* CXCR1 blockade selectively targets human breast cancer stem cells *in vitro* and in xenografts. J Clin Invest 120, 485–497 (2010). 20051626 10.1172/JCI39397 PMC2810075

[b33] Ning Y. *et al.* The CXCR2 antagonist, SCH-527123, shows antitumor activity and sensitizes cells to oxaliplatin in preclinical colon cancer models. Mol Cancer Ther 11, 1353–1364 (2012). 22391039 10.1158/1535-7163.MCT-11-0915

[b34] Sotlar K. *et al.* CD25 indicates the neoplastic phenotype of mast cells: a novel immunohistochemical marker for the diagnosis of systemic mastocytosis (SM) in routinely processed bone marrow biopsy specimens. Am J Surg Pathol 28, 1319–1325 (2004). 15371947 10.1097/01.pas.0000138181.89743.7b

[b35] Herold T. *et al.* Isolated trisomy 13 defines a genetically homogenous AML subgroup with high frequency of mutations in spliceosome genes and poor prognosis. Blood 124, 1304–1311 (2014). 24923295 10.1182/blood-2013-12-540716

[b36] Li Z. *et al.* Identification of a 24-gene prognostic signature that improves the European LeukemiaNet risk classification of acute myeloid leukemia: an international collaborative study. J Clin Oncol 31, 1172–1181 (2013). 23382473 10.1200/JCO.2012.44.3184 PMC3595425

